# Laparoscopic Adnexal Detorsion in a 20-Week Pregnant Patient: A Case Report and Literature Review

**DOI:** 10.1155/2019/1093626

**Published:** 2019-11-11

**Authors:** Rawad Halimeh, Serge Tomassian, Maria El Hage, Nicole Metri, Marianne Bersaoui, Rafi Daou, Elie Anastasiadis

**Affiliations:** ^1^Obstetrics and Gynecology Department, Saint George Hospital University Medical Center, Beirut, Lebanon; ^2^Faculty of Medicine and Medical Sciences, University of Balamand, El-Koura, Lebanon; ^3^Faculty of Medicine, University of London, St. Georges, Nicosia, Cyprus

## Abstract

Adnexal torsion is a cause of severe pelvic pain in reproductive aged women and during pregnancy. Adnexal torsion occurs when there is a complete turn of the ovary, tube, or both resulting in impaired blood flow to the ovary. The diagnosis of adnexal torsion is sometimes challenging due to the enlarged effect of the uterus, the displacement of abdominal and pelvic structures and the nonspecific symptoms in pregnancy. Therefore, prompt diagnosis is essential for better maternal and neonatal outcomes. The gold standard for confirmation and treatment of ovarian torsion is surgery. Laparoscopy and Laparotomy are surgical options with defined risks and benefits. Therefore, choosing the best surgical technique and surgical procedure are of utmost importance to decrease the chances of adverse events intra and postoperatively. Little literature exists regarding the laparoscopic approach of an ovarian torsion during the second trimester. Our case is a 20-week pregnant patient who had a 1080 degree rotation of the left adnexa. She required laparoscopy for adnexal detorsion and had good intraoperative, postoperative, maternal, and neonatal outcomes following management.

## 1. Introduction

The diagnosis of acute pelvic pain in pregnancy is often challenging [1]. Difficulties in diagnosing pelvic pain during pregnancy is mostly due to several pregnancy related factors [1]. These factors include the displacement of abdominal and pelvic structures by the enlarging gravid uterus, the nonspecific gastric symptoms, and the difficult abdominal examination in pregnancy [2]. Acute pelvic pain can present with nonspecific signs and symptoms that are associated with numerous conditions which can be grouped into different categories [1]. These categories include: (1) Non obstetrical-non gynecological causes such as urolithiasis, appendicitis, cholecystitis, and intestinal obstruction. (2) Obstetrical causes such as placental abruption, abnormally invasive placentas, and uterine rupture. (3) Gynecologic causes such as ovarian torsion, pain resulting from adnexal masses, and degeneration or necrosis of uterine fibroids [1]. Adnexal torsion occurs when the adnexa, ovary, or fallopian tube completes at least a full turn around a centre-line axis formed by the infundibulopelvic ligament and tubo-ovarian ligament [3]. This leads to an impaired venous flow, possibly followed by impaired arterial flow to the ovary. The lack of blood flow results in stromal edema, hemorrhagic infarction and necrosis respectively [4]. It accounts for approximately 3% of all gynecologic surgical emergencies in women and 80% of these instances happen in the reproductive age group [5]. Ovarian torsion is rare during gestation, developing in only 1 in 5000 pregnancies [6].

## 2. Case Presentation

A 32-year-old Lebanese female patient G2P0010 (spontaneous pregnancy), who was at 20 weeks and 2  days of gestation as per last menstrual period, presented to the emergency room for the evaluation of same day pelvic pain. The pain began 3 hours prior to presentation and was described as severe, stabbing in nature, nonradiating, and intermittent. The patient reported normal fetal movements and denied feeling contractions; she did not have any other associated symptoms.

Past medical and surgical histories were unremarkable. Patient denied cigarette smoking, alcohol intake, or any exposure to illicit drugs. She did not have any known food or drug allergies. Patient had a routine transabdominal obstetrical ultrasound at 12 weeks of gestation; No adnexal abnormalities were noted. The patient was only taking vitamins. Review of systems was negative as she denied any recent illness, fever, chills, night sweats, nausea or vomiting, suspicious food intake, or recent sick contact.

The patient had stable vitals in the emergency room with a blood pressure of 120/80 mmHg, heart rate of 80 beats per minute and a temperature of 37.3 degrees Celsius. Physical examination revealed positive active bowel sounds, no epigastric, left or right upper quadrant tenderness. Costovertebral angle signs were negative bilaterally, Mc Burney's point tenderness was negative, and Murphy sign was also negative. Cervical exam was done showing a cervix which was closed, posterior, and long. The patient had severe left lower quadrant tenderness with guarding that was not radiating on physical exam. No vaginal discharge was noted. Her pain was not relieved by intravenous pain medications.

Laboratory workup showed a complete blood count which was within normal ranges: WBC 10,400/mm^3^(Neutrophils 86.2%, Lymphocytes 9.3%), Hemoglobin 11.3 g/dL, Platelets 180,000/mm^3^, SGPT 45 U/L, GGT 7 U/L, PT-INR 1.1, PTT 29.86 seconds, C-Reactive Protein 0.9 mg/L; creatinine 0.51 mg/dL, Urine analysis: RBC 1/mm^3^, WBC 14/mm^3^, Urine culture: no growth after 48 hours of incubation.

**Figure 1:**
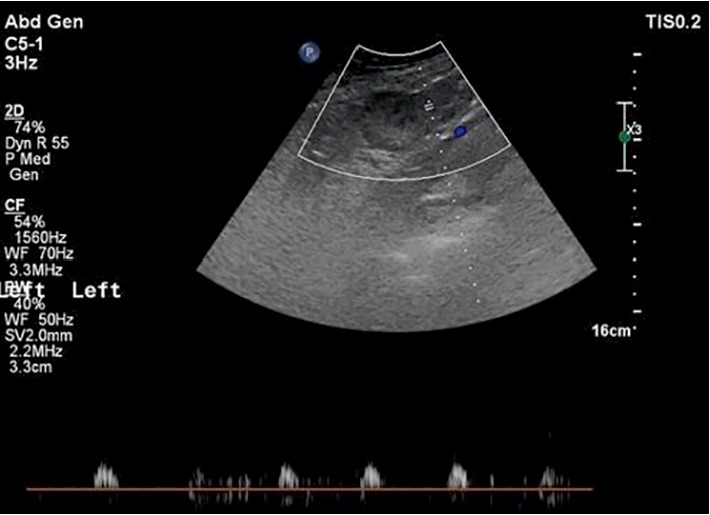
Absent Doppler flow to the enlarged left ovary.

After a primary assessment, the patient had an abdomino-pelvic ultrasound performed. Abdominal ultrasound was normal; however, the pelvic ultrasound showed an enlarged left ovary (59 × 51 × 54 mm) with diffuse hyperechogenicstroma, peripheral displacement of the follicles, and a hypoechoic rim and minimal fluid in the pelvis (Figure 1). Doppler was done and showed a nearly absent flow to the left ovary (Figure 1). This clinical scenario combined with the ultrasound result was compatible with the diagnosis of left sided adnexal torsion. Of note, fetal heart rate was normal at 157 beats per minute.

The decision for urgent laparoscopic detorsion was made to try to preserve ovarian function and prevent any adverse maternal or neonatal effects.

**Figure 2:**
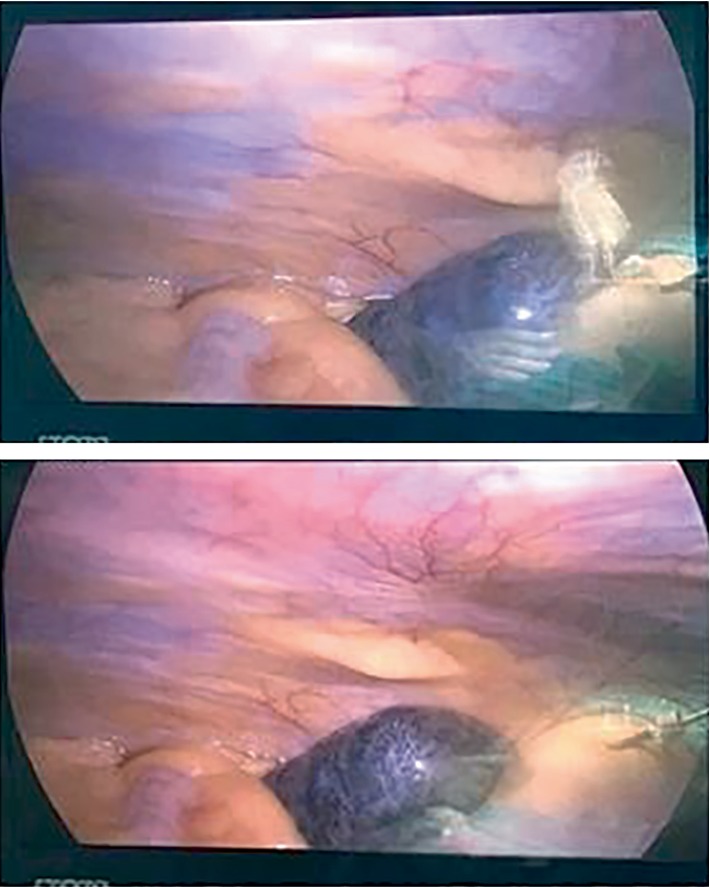
Dark purple discoloration of left ovary.

**Figure 3:**
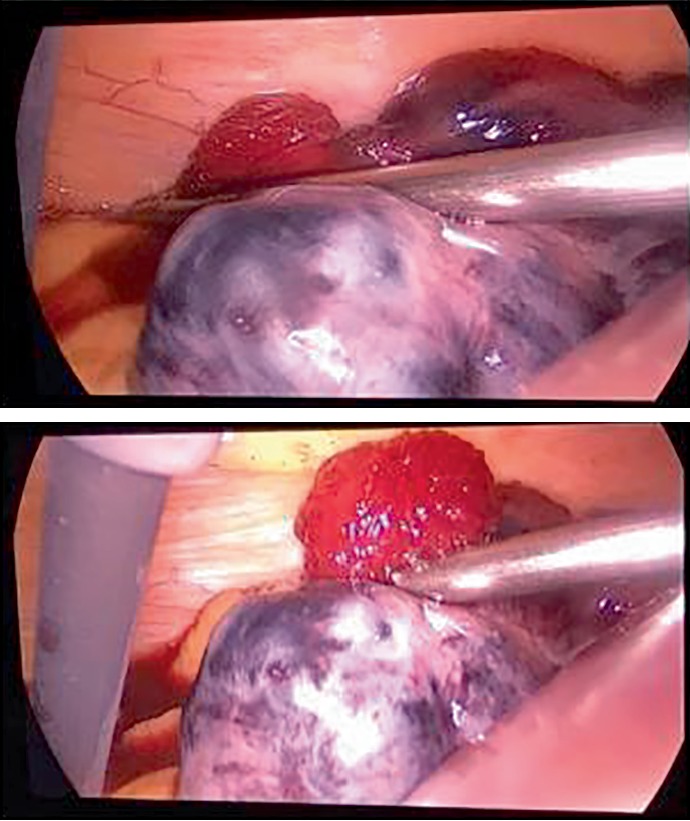
Gradual return of blood flow to the left ovary.

**Figure 4:**
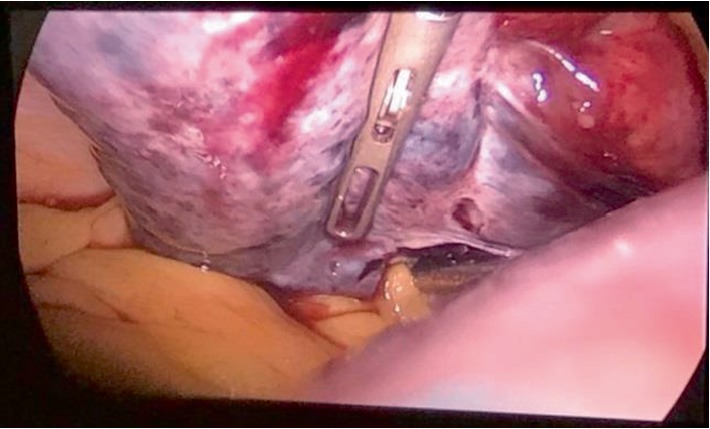
Complete return of blood flow to the left ovary.

Entry to the pelvis was challenging due to the enlarged uterus at 20 weeks of gestation; therefore, a Veres needle was introduced at Palmer's point. The abdomen was insufflated using CO_2_ with a pressure of 12 mmHg followed by insertion of trocars number ten at the supraumbilicus, and two number fives at the left lower quadrant and suprapubic area, respectively. A 20-week-sized uterus and the left adnexal torsion (ovary and tube) were visualized with dark purple discoloration of the ovary (Figure 2). Adnexal detorsion was completed with removal of the necrotic ovarian tissue and any blood clots. This was done with meticulous conservation of the ovarian parenchyma. A gradual return to normal coloration of the ovary was noted 6 minutes after detorsion (Figure 3). This was followed by a complete return to normal coloration after 10 minutes (Figure 4) with sufficient hemostasis assured. No intraoperative complications were encountered with a procedure time of 45 minutes. Postoperative Doppler ultrasound of the left adnexa showed normal flow. The patient presented for induction of labor at 39 weeks and 6 days of gestation following an uncomplicated pregnancy after surgery. She had an operative vaginal delivery (Forceps delivery) of a healthy baby boy (Weight 3810 g, Height 52 cm) with an Apgar of 9/10 and 10/10 at 1 and 5 minutes, respectively, and was discharged home on day 2 post-delivery.

## 3. Discussion

Most cases of ovarian torsion in pregnancy occur during the first trimester [5], but can also happen to a lesser degree in the second or third trimester [7]. According to Yen et al. 60% of ovarian torsion occurred between the 10th and 17th weeks of gestation, whereas only 5.9% took place after 20 weeks [8]. The risk factors for adnexal torsion include an enlarged ovary, ovarian tumors, ovarian hyperstimulation syndrome, and pregnancy [9–12]. Patients with ovarian torsion usually present with a sudden onset acute lower abdominal pain with guarding and other various nonspecific symptoms such as nausea and vomiting [13]. The pain can be constant or intermittent. There may be a history of transient episodes of pain, which indicate previous partial torsions since the ovary may torse and untorse with sudden change in position or activity. The intensity of pain is not severe in most of these intermittent episodes, which can occur for several days to months before admission. Occlusion of the vascular pedicle leads to hypoxia which causes this pain [14]. Since it is a surgical emergency, prompt diagnosis should be made to preserve residual function of the ovary [7]. The first step in diagnosing torsion is with imaging by an abdominal ultrasound [7]. In the case of ovarian torsion, almost all patients have an enlarged ovary on ultrasound; [7] “Whirlpool sign” is usually observed which is the main hallmark seen on imaging [15]. Doppler flow of the ovary may be normal or abnormal [16] and, therefore, does not improve accuracy of diagnosis due to the high false negative rates [6]. However, clinicians can predict the nonviability of the ovary with the presence or absence of arterial and venous blood flow on Doppler [17] making it a viable prognostic tool. Unfortunately, in some cases, the ultrasound result is equivocal and may not be sufficient for diagnosis of the torsion [18]. In such cases an MRI is helpful for diagnosis and can demonstrate the components of the mass in a clearer fashion than the ultrasound. An MRI can show the twisting of the pedicle [19] more so during the second and third trimesters due to the enlarged uterus [17]. Diagnosis of ovarian torsion with ultrasonography had overall accuracy of 96.0% with the sensitivity of 72.1% and the specificity of 99.6%, respectively [20]. Abnormal ovarian blood flow using Doppler was the most diagnostically accurate isolated sonographic sign with PPV 80.0% [21]. On the other hand, MRI has a sensitivity of 77.2% and a specificity of 86.1% for the diagnosis of adnexal torsion. The probability of adnexal torsion was increased six times in case of visualization of the whirpool sign and eight times higher in cases of visualization of tubal thickening [22].

The gold standard for confirmation and treatment of ovarian torsion is surgery whether laparoscopy or laparotomy [11]. The viability of the ovary is usually assessed by direct visualization of the ovary [11]. Dark (black or blue) and enlarged ovaries are usually associated with nonviability of the ovary; however, most of the times, they may retain function following detorsion [24]. Conservative surgical treatment is usually considered regardless of the color of the ovary since the clinical appearance does not correlate well with the residual function [23–25]. This logic counters the traditional method of managing ovarian torsion with removal of the adnexa [26], which was done due to the false belief that detorsion would pose a higher risk of thromboembolic events [27]. However, detorsion will carry a higher risk of recurrence [28].

### 3.1. Laparoscopy vs. Laparotomy for Management of Ovarian Torsion in Pregnancy

There are limited data available regarding the best surgical method for treating ovarian torsion in pregnancy. There are many maternal and fetal advantages of laparoscopy. The main maternal advantages are less operative blood loss, less postoperative pain, shorter hospital stay, and a decreased risk of preterm labor [29, 30]. However, fetal loss has always been a major concern when choosing the surgical approach. The main risk with laparoscopy is that it poses a risk on the fetus since it utilizes carbon dioxide for pneumoperitoneum, which can decrease the blood flow in the uterine arteries and maternal circulation causing intrauterine hypoxia [31, 32]. It can also increase the carbon dioxide absorption by the fetus leading to fetal acidosis [33]. Thus, having an intraperitoneal pressure of less than 12 mmHg and performing laparoscopy with an estimated time of less than thirty minutes is considered safe in pregnancy [34].

Nevertheless, the laparoscopic technique during pregnancy is challenging and risky [35]. Visualization of the surgical field may be compromised by the increasing size of the gravid uterus, which could lead to Trocar injury to the uterus [35, 36]. Therefore, either Veres needle insertion or Hasson technique (open technique) could be used depending on the surgeons' skills and preference. Trocar sites should be limited to subxiphoid, left upper quadrant, or right upper quadrant access points which will aid in avoiding uterine injury [35]. In addition, a sufficient distance between the uterus and the tip of the laparoscope can be obtained by placing a supraumbilical primary trocar allowing for an ideal visual field [35] especially during the second and third trimesters. In our case, it was difficult to place the trocar in the infrumbilical site due to the enlarged uterus reaching past the umbilicus, so we opted for the supraumbilical site. It is worthy to note that the uterus should never be manipulated by a transcervical device [35].

Moreover, the positioning of the patient is an important factor to consider in laparoscopic ovarian detorsion. As previously mentioned, carbon dioxide in the pneumoperitoneum affects the maternal venous blood flow causing a decrease in the preload and an increase in the afterload and systemic vascular resistance. Thus, placing the patient on her left lateral side with a 30-degree angle in Trendelenburg position would relieve the compression on the inferior vena cava, increase the venous return to the maternal heart, normalize the pulmonary pressure, and increase the cardiac output during surgery [37]. It would also help us get a better visual field through displacing the abdominal structures cephalad.

### 3.2. Role of Gaseless Laparoscopy for Management of Ovarian Torsion in Pregnancy

The gasless laparoscopy, a surgery done with a vertical midline umbilical skin incision is made with the abdominal wall lifted anteriorly to achieve exposure [38]. A laparoscope is then introduced into the abdominal cavity for inspection [38].

Gasless laparoscopy was shown to be a safer approach than CO2 laparoscopy, especially in patients with a history of previous abdominal surgery, and resulted in excellent cosmetic results since the single surgical wound was limited to the umbilicus [38]. Problems that could arise in CO2 laparoscopy such as bradyarrhythmias, venousstasis, gasembolism, subcutaneous emphysema and hypercarbia were not observed in gasless laparoscopy [38]. The CO2 laparoscopy resulted in an increase in elevation in the central venous pressure, respiratory function, and stress response in contrast with gasless laparoscopy [39]. Postoperative recovery time of borborygmus and the incidence of postoperative nausea, vomiting, and shoulder pain were much lower in gasless than CO2 laparoscopy [39].

Moving on to the postoperative and obstetric care, it is recommended that fetal heart rate is to be measured and documented postoperatively alongside monitoring of uterine contractions. They will be used to assess for early signs of preterm labor [37]. However, the systematic use of tocolysis is to be avoided [37], unless signs of preterm labor are evident. Intraoperative pneumatic compression devices are to be used to prevent any thromboembolic event during surgery especially since the patient is already at a high risk of hypercoagulable diseases during her pregnancy. Early ambulation is to be advised as well to decrease the risk of deep vein thrombosis [37].

## 4. Conclusion

It is becoming evident that laparoscopic surgery for ovarian torsion during pregnancy is safe and feasible, does not increase maternal or fetal complications [40], and shows no significant difference regarding gestational age at delivery or mode of delivery [40]. Therefore, laparoscopy can be done throughout all trimesters with no differences in outcomes from laparotomy. However, the complexity of the laparoscopy increases with the increasing gestational age so it should only be done by experienced surgeons who take into careful consideration the operative time, the intraperitoneal pressure, the positioning of the patient, and the placement of the trocars to decrease risks and complications [37]. In our case, gasless laparoscopy was not done mainly because of the safety of CO2 laparoscopy when done with a short surgical time, appropriate pressure, and expertise.
